# Review of the Literature: Surgery Indications for Fuchs’ Endothelial Corneal Dystrophy

**DOI:** 10.3390/jcm14072365

**Published:** 2025-03-29

**Authors:** Moïse Tourabaly, Juliette Knoeri, Cristina Georgeon, Vincent Borderie

**Affiliations:** Centre Hospitalier National d’Ophtalmologie des 15-20, GRC32 Sorbonne Université, 28 rue de Charenton, 75571 Paris, Francevincent.borderie@upmc.fr (V.B.)

**Keywords:** Fuchs’s dystrophy, DMEK, DSAEK, preoperative acuity, SD-OCT

## Abstract

**Objectives:** To provide an overview of the preoperative indications for endothelial graft in patients with Fuchs endothelial corneal dystrophy (FECD). **Methods:** A comprehensive database search without date restrictions was performed in PubMed. Keywords included Descemet membrane endothelial keratoplasty (DMEK), Descemet stripping automated endothelial keratoplasty (DSAEK), corneal keratoplasty, preoperative visual acuity, preoperative central corneal thickness, and densitometry. Articles aiming to describe or evaluate preoperative indications for endothelial keratoplasty were considered eligible and were included in this review. **Results:** The indications for surgery in FECD are disparate between the different studies. The tendency is to operate on patients earlier to obtain a better postoperative visual acuity at 1 year. The surgical decision is based on a number of arguments (visual acuity, CCT, densitometry). A preoperative visual acuity worse than 20/40 is generally considered a surgical indication for DMEK, based on current literature. **Conclusions:** Surgical decisions for Fuchs’ dystrophy should be individualized, guided by preoperative visual acuity, corneal OCT, and advanced imaging, with future risk scores potentially refining the timing of intervention to optimize outcomes.

## 1. Introduction

Fuchs’ endothelial corneal dystrophy is currently the first indication for corneal transplantation in Western countries. This increase in the number of keratoplasties performed for Fuchs dystrophy may be the result of population aging and the development of endothelial keratoplasty, which features several advantages over penetrating keratoplasty. In addition, the level of visual impairment at the time of the transplantation decision, i.e., the preoperative visual acuity, tends to be lower when endothelial keratoplasty techniques are implemented. As age is a strong risk factor for Fuchs’ dystrophy, cataract is often associated with Fuchs’ dystrophy, and many patients are considered for a triple procedure combining phacoemulsification, intraocular lens implantation, and endothelial keratoplasty. In such cases, the visual recovery may result from cataract surgery, keratoplasty, or both.

Despite the good results of endothelial keratoplasty, a transplantation procedure carries several risks in the short, mid, and long term that may threaten the patient’s vision. A benefit/risk ratio has to be assessed for any Fuchs dystrophy patient with impaired vision to determine whether he/she would need a keratoplasty, a keratoplasty combined with cataract surgery, cataract surgery alone, or no surgery. Keeping in mind the primary principle of medicine (*primum non nocere*), any unnecessary surgery should be avoided.

Our objective was to review the rationale for performing endothelial keratoplasty in Fuchs dystrophy to provide physicians with a few guidelines to make the appropriate decision.

## 2. Materials and Methods

This is a systematic review following the Preferred Reporting Items for Systematic Reviews and Meta-Analyses (PRISMA) guidelines.

### 2.1. Eligibility Criteria

We included all studies assessing preoperative indications for Fuchs’ dystrophy keratoplasty. Keywords included Descemet membrane endothelial keratoplasty (DMEK), Descemet stripping automated endothelial keratoplasty (DSAEK), corneal keratoplasty, preoperative visual acuity, preoperative central corneal thickness, and densitometry.

### 2.2. Data Sources

We conducted systematic searches in the PubMed database from inception to 31 December 2024.

### 2.3. Selection Process

References identified by the electronic search were entered and managed into Zotero 7.0 software, and duplicates between databases were identified and removed. One author (M.T.) screened titles and abstracts first. Then, 2 authors (M.T and J.K.) independently read the full text, applying eligibility criteria.

### 2.4. Data Extraction

Two authors (M.T. and J.K.) independently extracted data from each study based on a data extraction form and entered data into Microsoft Excel. A third author (V.B.) was asked to review discrepancies not solved by discussion to reach a consensus.

### 2.5. Data Items

We collected the following items:

1. Title, authors, year of publication, and journal;
2. Study design: RCT/nonrandomized, retrospective, or prospective cohort study;
3. Country;
4. Participants: total number of eyes, age, sex, means of diagnosis, criteria for diagnosis, surgical indications (Fuchs’ dystrophy and bullous keratoplasty), surgical techniques, preoperative visual acuity, CCT preoperative, 1-month postoperative CCT, mean reduction in CCT (%).

## 3. Physiopathology

### 3.1. Role of the Corneal Endothelium

The corneal endothelium is a single cellular layer, with a thickness between 1.5 and 2.5 µm. It maintains the transparency of the cornea by controlling the quantity of water in the stroma (which is the function of the endothelial pump). It also acts as a barrier at the entrance of the aqueous humor in the cornea and controls the exits of water from the stroma towards the aqueous humor (pump Na^+^/K^+^, ATPase) [1].

### 3.2. Endothelial Density

The average density of endothelial cells for a one-month-old baby is 6000/mm^2^ [2], and decreases to 3500/mm^2^ by the age of 5 [3]. The central average cellular density decreases by 0.6% each year [4].

The average population aged over 50 has an average cellular density between 2700/mm^2^ and 2800/mm^2^ [5]. This density decreases dramatically with age. At 85, the average density drops to 2300 cell/mm^2^ [6]. The function of the endothelial pump is maintained as long as the endothelial density remains superior to 450–500 cells/mm^2^.

### 3.3. Fuchs’ Dystrophy and Endothelium

Fuchs’ dystrophy is a primary disorder of the endothelial-Descemet’s corneal layer originally described by Doctor Ernst Fuchs in 1910 [7]. It is characterized by the formation of central droplets in Descemet’s membrane (named cornea guttata), which could evolve towards intra-corneal edema. Fuchs’ dystrophy is also characterized by a reduction of the endothelial density shown by confocal microscopy of approximately 45 to 59% compared with the general population [8].

The endothelial layer is thinner in Fuchs’ dystrophy and can go up to 0.06 µm [9].

In Fuchs’ dystrophy, the endothelial function of the pump between the stroma and the aqueous humor increases in the early phase [10], and then deteriorates with the evolution of the disease. It is when the barrier function of the endothelium is altered that intracorneal edema appears and a loss of visual acuity arises [11].

Cellular apoptosis and the activation of senescence have been proven in Fuchs’ dystrophy. A study has shown that during analysis using the test of nuclear labeling 2.6% of endothelial cells per sample proved to be apoptotic among patients who had Fuchs’ dystrophy, against 0.2% amongst the control group [12].

### 3.4. Fuchs’ Dystrophy and Descemet’s Membrane

Descemet’s membrane is not visible in specular microscopy in young patients. With age, it becomes more and more visible and corresponds in specular microscopy to an acellular layer, which is between the posterior stroma and the endothelium [13].

In Fuchs’ dystrophy, Descemet’s membrane is thickened, and the guttae appear as luminous spots, each being surrounded by dark circular rings of 15 to 40 μm in diameter in confocal microscopy [8].

Descemet’s membrane corresponds to an entity of two layers of acellular collagen of approximately 4 to 6 µm in histology among healthy subjects [9]. In Fuchs’ dystrophy, we notice the emergence of a third layer corresponding to modified collagen VIII and of a fourth layer made up of an extracellular fibrous matrix [14]. This third cellular layer contains the guttae, and the fourth layer covers these guttae.

### 3.5. Fuchs’ Dystrophy and Genetics

Fuchs’ dystrophy is a hereditary pathology with a dominant autosomal transmission with a high level of penetrance and a variable expression [15]. The pathophysiology of this dystrophy results from interactions between genetic and epigenetic factors affecting the corneal endothelial cell (oxidative stress, hormonal factors, accumulation of proteins in the cell with an abnormal tertiary structure that is not folded, mitochondrial dysfunction, and mitochondrial autophagy). A variation at the level of transcription factor gene 4 (*TFC4*) is often associated with Fuchs’ dystrophy [16]. A study has found that a repetition of >50 of the trinucleotide TGC was strongly associated with Fuchs’ dystrophy [17]. It is the most frequent of the genetic abnormalities associated with the usual late form of the dystrophy. A mutation of the gene *COL8A2* is associated with an early form of Fuchs’ dystrophy [18]. Numerous genes have been involved in the physiopathology of the late form: *COL8A2*, *DMPK*, *SLC4A11*, *ZEB1/TCF8*, *LOXHD1*, *AGBL1*, *KANK4*, *LAMC1*, *ATP1B1*, *RAD51*, *FEN1*, *XRCC1*, *NEIL1*, *TGFBI*, *CLU*, *PITX2*, *PTPRG*, *FASLG*, *KCNJ13*.

## 4. Epidemiology

The prevalence of Fuchs’ dystrophy and *cornea guttata* depends on age and gender (mostly female). Data accumulated over a seven-year study has shown that the incidence of *cornea guttata* was the highest amongst patients aged 55 to 64 [19].

Hyperopia (+2.48 diopters versus −0.31 diopters), a low axial length (22.1 mm versus 23.4 mm), and a narrow anterior chamber (2.2 mm versus 2.7 mm) have been associated with Fuchs’ dystrophy. However, the low number of observations does not allow us to conclude for sure the existence of a true link between them [20,21].

The association between ultraviolet radiation and Fuchs’ dystrophy remains debatable. However, a study on mice has shown that ultraviolet radiations of type A (UVA) bring about an oxidant stress at the level of the DNA and hence an alteration of the endothelial cells. It is important to note that for women, the activation of the UVA rays is due to the CYP1B1, an enzyme that transforms estrogens into metabolites that deteriorate the DNA [22].

## 5. Surgical Techniques

### 5.1. Descemet Stripping Automated Endothelial Keratoplasty (DSAEK)

DSAEK is an endothelial lamellar graft corresponding to the corneal stroma-Descemet’s membrane and the corneal endothelium.

In the operating theater, the grafts are mounted on an artificial anterior chamber. After complete removal of the epithelium, a lamellar cut is performed with a microkeratome depending on the thickness of the graft. The graft is then trephined. A descemetorhexis is carried out either under viscoelastic or under air. The graft is then inserted either manually or with the help of a device such as an injector and applied to the posterior face of the patient’s cornea and maintained with an air bubble or a mixture of air and SF6 (sulfur hexafluoride).

### 5.2. Descemet Membrane Endothelial Keratoplasty (DMEK)

DMEK corresponds to a posterior lamellar graft made up of Descemet’s membrane and corneal endothelium.

The corneal graft is positioned in the base of the trephine, and then the graft is marked with trypan blue. The trabecular meshwork is dissected first in order to have access to Descemet’s membrane and to detach it at the level of Schwalbe’s line. The detachment of Descemet’s membrane is achieved with a saline solution (BSS) and closure over 360° to limit the enlargement of an eventual splitting line. Once all adherences have been cleared, the endothelio-descemetic layer is peeled in a centripetal manner under total immersion in BSS, hence limiting adherences to the maximum. The graft is marked depending on the surgeon’s practices. It is then totally detached and put into a glass injector.

A descemetorhexis is performed under viscoelastic or air on the patient, and then the graft is carefully injected to avoid a tear in the graft. Various manipulations are also performed to unroll it completely (with or without contact), orienting the stromal side towards the top and centering it carefully with respect to the descemetorhexis area. The correct positioning of the graft is checked either by the marking on the graft or by using an OCT microscope during surgery [23]. The tamponade of the graft is identical to that of the DSAEK technique. However, a study shows that SF6 diluted to 20% with air decreases the rate of post-surgery rebubbling compared to air tamponade [24].

**Figure 1:**
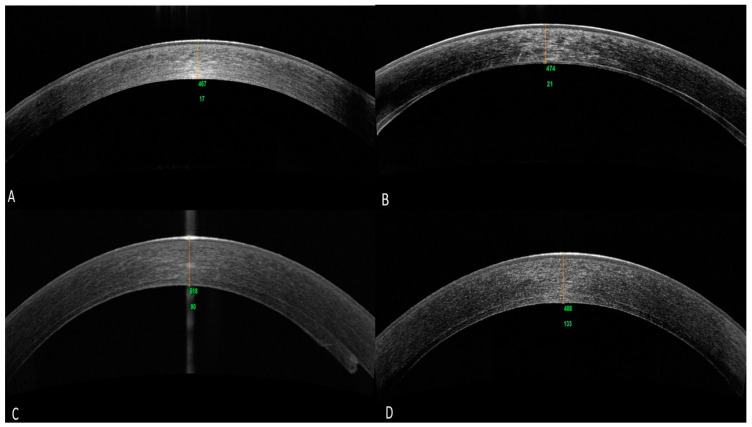
Spectral-Domain Optical Coherence Tomography view of the different types of endothelial grafts. Horizontal cross-sections of the corneal apex. (**A**) DMEK (graft thickness not measurable: posterior hyperreflective line (endothelo-Descemet’s membrane) < 15 µm); (**B**) nanothin DSAEK (15 < graft thickness < 50 µm); (**C**) ultrathin DSAEK (50 ≤ graft thickness < 100 µm); (**D**) DSAEK (graft thickness ≥ 100 µm). The yellow horizontal line represents the reference axis along which the thickness of the endothelial graft and the adjacent host cornea were assessed on cross-sectional OCT images. Source: Georgeon C., Tourabaly M.

Figure 1 shows the SD-OCT aspect in horizontal section at the corneal apex of a DMEK and DSAEK with grafts of various thicknesses.

### 5.3. Current Trend for Endothelial Keratoplasty

Endothelial transplants have seen a boom these last years with respect to penetrating grafts. Fuchs’ dystrophy is the first indication of a corneal graft before keratoconus [25]. Fuchs’ dystrophy is the first indication of endothelial grafts ranging from 85.9% to 88.8% according to several studies [26], followed by bullous keratopathy, whose main etiology is endothelial decompensation following cataract surgery [25,27]. In 2012, a worldwide survey carried out in 116 countries showed that most corneal grafts (39% out of 184,576 grafts) only targeted the treatment of Fuchs’ dystrophy [28].

DMEK has known considerable growth for several years at the expense of DSAEK [29]. In fact, DMEK allows for an improvement in the speed of visual recovery and a better final visual acuity, as well as a lowering of the risk of rejection.

Compared to DSAEK, DMEK surgery is cheaper and would allow 0.4 extra quality years of life over a 15-year period [30].

## 6. Preoperative Assessment of Fuchs’ Dystrophy Patients

### 6.1. Symptoms

The first symptoms reported by patients affected with Fuchs’ dystrophy are morning fog and photophobia, followed by a lowering of visual acuity [31]. The limitations of activities result from photophobia or fluctuations in visual acuity during the day, with an improvement of vision in the day [32]. Patients with Fuchs’ dystrophy with no corneal edema may present a decrease in the quality of vision particularly linked to light diffraction provoked by the damaged endothelium with guttae formation [33].

A team has developed a specific reproducible questionnaire that allows the evaluation of the visual handicap amongst patients affected by Fuchs’ dystrophy: the V-FUCHS questionnaire has seven items concerning visual acuity and eight items on photophobia and the variation in visual acuity during the day [34]. A German team has also used this questionnaire and finds it to be a reliable tool, reproducing the visual handicap as felt by the patient [35].

### 6.2. Visual Acuity

#### 6.2.1. Preoperative Visual Acuity in Studies Reporting the Results of Keratoplasty in Fuchs’ Dystrophy

There is a trend toward higher preoperative visual acuity in DMEK eyes compared with DSAEK and PK eyes [36], and a trend toward better preoperative vision in more recent studies (Table 1). A meta-analysis reported an average preoperative visual acuity of 0.48 LogMAR amongst 648 patients undergoing DMEK [37].

#### 6.2.2. Relationship Between Preoperative Visual Acuity and Visual Recovery

A recent study has shown a correlation between preoperative visual acuity and postoperative visual acuity in the first year after surgery. Indeed, a visual acuity inferior to 20/100 leads to a lower visual recovery. The chances to obtain a 20/25 visual acuity after surgery are 40% for a preoperative visual acuity of 20/200, 50% for a preoperative visual acuity of 20/60, and higher than 60% for a preoperative visual acuity of 20/40 [53].

### 6.3. Slit-Lamp Examination

Table 2 shows the historical classification based on the examination with a fine slit with strong magnification (×40) quantifying endothelial guttae [54].

This classification shows the morphological details with the distribution of endothelial guttae but does not reflect the corneal endothelial function. This method remains subjective and variable depending on the observer. A study has shown the correlation between two observers who have evaluated clinically Fuchs’ dystrophy according to Krachmer’s classification (0 to 6). A good match between the two observers has been obtained in only 44% [55].

Retro-illumination photography allows a more objective analysis of the distribution of endothelial drops [56]. However, this method is not easy to achieve generally, and it does not permit to determine the presence and the severity of corneal edema.

### 6.4. Optical Coherence Tomography

The anterior segment optical coherence tomography (AS-OCT) is a breakthrough in the imaging of many disorders of the anterior segment and the cornea [57]. It allows precise quantification of the central and peripheral corneal thicknesses and a follow-up of the evolution of endothelial dystrophy. The AS-OCT, and specifically, the Spectral-Domain OCT, is a routine examination in patients with endothelial disorders. Table 3 summarizes the largest clinical studies reporting preoperative and postoperative central corneal thickness in DMEK procedures. One of the surgical indications for endothelial keratoplasty is corneal edema. The best examination to diagnose subclinical corneal edema remains Spectral-Domain AS-OCT when compared with ultrasound pachymetry [58]. In addition, the corneal transparency can be assessed from the OCT images by measuring the photon mean free path in the corneal stroma [59].

A preoperative CCT was found as a prognostic factor for the success of DMEK surgery. Eyes with CCT < 625 μm had a visual acuity at 12 months of 0.05 ± 0.07 LogMAR compared with 0.13 ± 0.11 LogMAR (*p* = 0.002) for eyes with a preoperative CCT > 625 μm [49].

#### Ratio Between Central and Peripheral Corneal Thicknesses

It has been shown that the corneal thickness ratio between the center and the 4 mm peripheral zone is an objective examination, which can be repeated routinely and which also evaluates the severity of the disease, with a ratio of 1.03 ± 0.07 and 0.95 ± 0.07, respectively, in the advanced and moderate stages compared with 0.87 + 0.05 for normal corneas [55].

### 6.5. Elevation Corneal Topography with Scheimpflug Camera

A recent study has shown the following three signs of subclinical intracorneal edema with the Scheimpflug topographer among patients with cornea guttata visible with a slit lamp but without subclinical signs of corneal edema [61]:

- Loss of parallelism or regularity of isopatches;
- Displacement of the finest point;
- Posterior focal depression.

**Figure 2:**
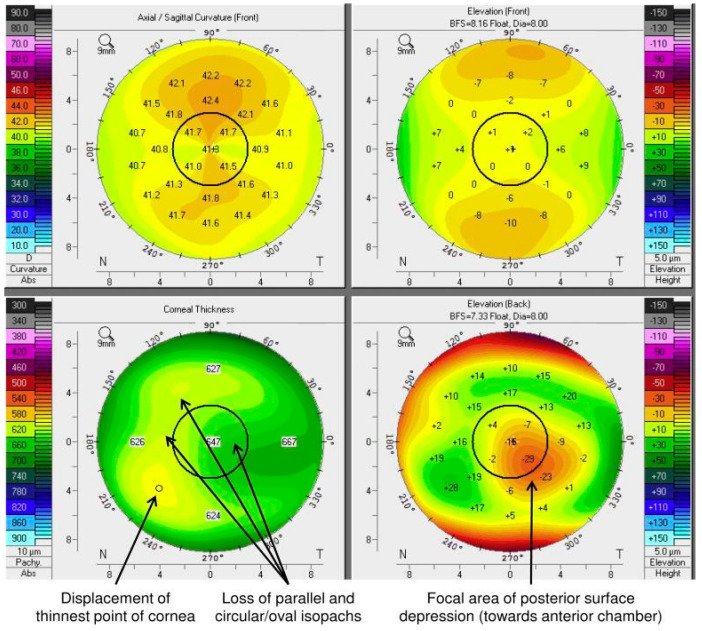
Tomographic characteristics with Scheimpflug’s technique of a subclinical corneal edema.

Scheimpflug tomography can detect subtle structural changes suggestive of subclinical corneal edema in patients with Fuchs’ dystrophy, as illustrated in Figure 2.

These signs were found as risk factors in multivariate analysis independently from the progression of Fuchs’ dystrophy assessed with CCT, with a cumulative risk of progression and surgery over 5 years of 7%, 48%, and 89% when one, two, or three of the signs were present, respectively [62].

The subjective interpretation of the images of the Scheimpflug technique is reproducible despite diurnal variations linked to the physiopathology of Fuchs’ dystrophy (diurnal fluctuations in corneal hydration) [63].

### 6.6. Corneal Densitometry

Corneal densitometry is a tool allowing the objective evaluation of corneal transparency. It uses Scheimpflug’s technology, which permits an analysis of the transparency of the different corneal layers. Patients suffering from Fuchs’ dystrophy show higher corneal densitometry compared to the general population [64].

Patients with endothelial dysfunction show a disorder of the collagen matrix due to corneal edema. Corneal opacity, which can appear with the evolution of the disease, provokes an increase in the diffusion of light, which corresponds clinically to corneal haze. Patients with Fuchs’ dystrophy who have corneal edema show higher densitometry compared to the same types of patients with no edema [65].

A study has highlighted the change in corneal densitometry after DMEK [60]. These patients benefit from an improvement in their corneal densitometry after the endothelial keratoplasty, with no correlation between the improvement in corneal edema assessed by CCT and the decrease in densitometry. Post-surgery, there is a significant correlation between corneal densitometry and the best-corrected visual acuity, but there is no correlation between the CCT and corneal densitometry. The correlation between corneal densitometry and visual acuity post-surgery is maximum in the anterior layers of the cornea and the central zone of the cornea.

However, no differences between DMEK and UT-DSAEK have been observed in the level of the corneal densitometry, which could suggest that other factors, such as optical aberrations, can affect the final visual result [66].

Hence, an increase in corneal densitometry would be in favor of endothelial keratoplasty in Fuchs’ dystrophy eyes.

### 6.7. Specular Microscopy

In Fuchs’ dystrophy eyes, the guttae are visualized as dark events with a clear center, completely concealing the cells that cover them. The cells located on the guttae are not in the specular reflection plane, which prevents them from reflecting light. Changes in the endothelial cell mosaic morphology, such as pleomorphism (a decrease in the hexagonal cells), polymegathism (anisocytosis), and a decrease in the endothelial cell density, can be observed outside the guttae [67].

**Figure 3:**
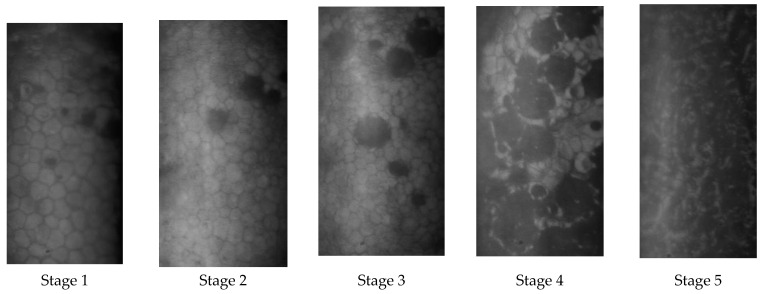
Specular microscopy of the different stages of cornea guttata (Laing’s classification).

Table 4 shows Laing’s classification of cornea guttata assessed with specular microscopy [68], and Figure 3 shows the different aspects of the different stages in specular microscopy.

Whether contact or non-contact, the specular microscopy technique does not influence the precision of endothelial cell count, be it central or peripheral [69].

Corneal edema in patients with an indication of endothelial keratoplasty prevents endothelial cell counting by specular microscopy. The endothelial cell density of the donor tissue for endothelial keratoplasty varies according to studies: 2704.3 ± 237.6 cells/mm^2^ [53], 2553 ± 194 cells/mm^2^ [70], 2602 ± 243 cells/mm^2^ with an endothelial loss of 30 to 40% during the first year [48]. A study has shown that 8 years after DMEK, for every additional 100 cells/mm^2^ in the preoperative graft cell density, the postoperative endothelial cell density rose by 86 cells/mm^2^ [71].

Therefore, specular microscopy seems more appropriate in the follow-up of patients with a non-decompensated endothelial function.

### 6.8. In Vivo Confocal Microscopy

In vivo confocal microscopy is a useful contact imaging modality that allows the visualization at a cellular and microstructural level of the corneal nerves [72]. In Fuchs’ dystrophy eyes, it allows visualization of the same alterations as described in specular microscopy but with higher resolution [73]. The density of the subbasal nerve plexus is decreased, and this appears from the early stages of Fuchs’ dystrophy [74].

In the advanced stages of Fuchs’ dystrophy, the endothelial periphery as assessed with in vivo confocal microscopy is a strong predictive marker of the severity of the disease compared with other usual biomarkers of the disease [75].

### 6.9. Aberrometry

The evaluation of anterior and posterior corneal optical aberrations (from images obtained with a Scheimpflug camera) in Fuchs’ dystrophy shows an increase in higher-order aberrations (HOA) from the early stages of the disease (before the appearance of corneal edema) [76]. The ultra-structural modifications of the anterior cornea, such as the loss of keratocytes and sub-epithelial fibrosis, contribute to the increase in HOA [77].

The increase in posterior corneal optical aberrations is caused by guttae, which create a non-regular posterior corneal surface [78].

HOAs of the anterior or posterior cornea alter the spreading function of the retinal image and reduce visual acuity [79].

## 7. Surgery-Associated Risks in Fuchs’ Dystrophy Patients

### 7.1. Cataract Surgery

#### Endothelial Decompensation Following Phacoemulsification/Descemet’s Membrane Detachment

Patients with endothelial disease prior to cataract surgery are most likely to develop endothelial decompensation after phacoemulsification [80].

Descemet’s membrane detachment is a rare complication during cataract surgery. The literature provides a small case series, and the most efficient management is air tamponade [81].

### 7.2. Keratoplasty

#### 7.2.1. Keratoplasty Complications Limiting Graft Survival

A nationwide study of 94,829 endothelial keratoplasty procedures showed that the overall 90-day cumulative incidence of postoperative endophthalmitis and choroidal hemorrhage was 0.03% and 0.05%, respectively, and the overall 1-year cumulative rates of retinal detachment, infectious keratitis, and cystoid macular edema were, respectively, 1.0%, 0.8%, and 4.1% [82].

Repeated EK grafts have the worst outcome with graft failure ranging from 12.5% to 24% [71,83]. Whereas mid-term follow-up studies tend to demonstrate satisfying survival of the donor corneal endothelium after endothelial keratoplasty, a recent study demonstrated lower endothelial survival after DMEK and DSAEK compared with PK [84].

The re-bubbling rate after DMEK ranges between 12.6% for a study of 857 DMEK to 19% in the Dutch cohort [85,86].

Re-bubbling increased the risk for endothelial cell loss but did not influence the postoperative visual acuity and the rate of graft failure [87,88].

#### 7.2.2. Graft Rejection

The overall DMEK survival probability in a study was 96% at 5 and 8 years postoperatively.

For DSAEK, a 3-year graft survival rate of 87% to 97% has been reported [89,90]; for DSEK, a 5-year survival rate of 93% has been reported [91]. After PKP, survival rates may vary from 75% to 95% at 3 and 5 years [89,91,92].

UT-DSAEK is very efficient in the management of primary DMEK graft failure, allowing visual rehabilitation, which is comparable with that of repeat DMEK. Twelve months after UT-DSAEK, BSCVA was ≥20/25 in 12/13 eyes [93].

#### 7.2.3. Glaucoma

In the US nationwide study, the probability of glaucoma surgery among patients with pre-existing glaucoma was 29% vs. 8% among those without pre-existing glaucoma at 8 years, and with a glaucoma surgery rate of 7.6%, 12.2%, and 13.8% after 1, 5, and 8 years, respectively [82].

#### 7.2.4. Increased Endothelial Cell Loss Leading to Late Endothelial Failure

Re-bubbling was found to be a key factor for ECD loss at 1 year after DMEK compared to patient-related factors, type of tamponade (air or 20% sulfur hexafluoride gas), and type of surgery (triple DMEK or DMEK alone) [94].

However, another study shows that the combined triple-DMEK procedure resulted in significantly greater loss of endothelial cells than DMEK-only surgery at both 1 month and 1 year [95], ocular trauma being one of the main causes of poorer visual outcomes after PK [96].

#### 7.2.5. IOL Opacification

IOL opacification, irrespective of the manufacturer or the exact composition of the hydrophilic lens material, increases after the instillation of exogenous material such as air or gas into the anterior chamber [97].

High-resolution OCT can visualize IOL opacities, and the amount of opacification correlates well with the stray light induced by the lens [98].

**Table 4:** Laing’s classification of Fuchs’ dystrophy in specular microscopy [98].

Stage	Guttae	Size of Guttae	Endothelium Around the Guttae	Remote Endothelium
1	Isolated with well-defined, clear central spot	<1 cell	Normal	Normal
2	Isolated	1 cell	Elongated cells forming a rosette with a blurred outline around the guttae	Normal
3	Beginning of confluence Regular round guttae with a central spot well defined as round or oval Irregular guttae with a central spot with ill-defined limits and of variable intensity	5 to 10 cells	Rosettes	Normal
4	Confluent, multilobed images with several clear spots Isolated guttae irregularly distributed	Large areas	Abnormal	Abnormal
5	Inverted endothelial reflection with clear outlines a lot more brilliant than the normal cellular surface surrounding black zones	Large areas	Not visible	Not visible

#### 7.2.6. Ocular Surface Disorders

Quality of vision (glare, hazy vision, daily vision fluctuations) has been shown to be improved after DMEK surgery [99].

DMEK does not impact corneal nerves. Therefore, dry eye parameters are not impaired by surgery. However, superficial corneal irregularity can prolong the surgery and can cause a lesser improvement of the best visual corrected acuity [100].

Refraction issues (astigmatism, HOA; EK versus PK)

DMEK has been shown to change the posterior astigmatism, which has to be accounted for in the IOL power calculation before a triple procedure [101].

High-order aberrations assessed by aberrometry were comparable between DMEK and DSAEK (ranging from nanofin to conventional DSAEK) [50].

A study shows the total HOAs after DMEK improved from 1.94 ± 1.05 μm preoperatively to 1.05 ± 0.16 μm at 12 months postoperatively (*p* < 0.001), but it is still significantly higher than the normal population [102]. PK has been shown to feature higher HOAs postoperatively than DSAEK: 2.9 ± 1.9 mm versus 1.9 ± 2.8 mm in the DSAEK-operated eyes [103].

## 8. Benefits and Risks of the Triple Procedure (Endothelial Keratoplasty Combined with Cataract Surgery)

A central corneal thickness > 640 µm in patients with Fuchs’ dystrophy is a risk factor for endothelial decompensation after cataract surgery [62,104,105].

When a cataract is clinically identified in a patient with endothelial dysfunction and corneal edema, the surgical decision is to perform a triple procedure combining phacoemulsification with endothelial keratoplasty and posterior chamber intraocular lens implantation.

### 8.1. Cataract Surgery in Patients with Endothelial Dysfunction

#### 8.1.1. Cataract Surgery Before Endothelial Keratoplasty

In a population without endothelial dystrophy, cataract surgery results in an average endothelial cell loss of 346 cells/mm^2^ [106].

The difference in endothelial cell loss after cataract surgery in patients with a low endothelial count (500 to 1000 cells/mm^2^) is comparable to the endothelial cell loss after cataract surgery in patients with a normal endothelial count with a mean cell loss of 5.1% and 4.2%, respectively [107].

Endothelial keratoplasty in phakic patients leads to an acceleration in the development of a cataract in the first year for DMEK [108] and DSEK [109]. However, in a series of 256 phakic patients operated on with DMEK, only 4% of patients needed cataract surgery in the six months following transplantation [26], contrary to another small study in which 33% of 49 eyes needed this surgery [108].

#### 8.1.2. Triple Procedure Versus Endothelial Keratoplasty in Phakic Patients

The question of the benefit of the triple procedure is a concern if the lens is clear, particularly with young patients.

National studies show a proportion from 20 to 25% of DMEK in phakic patients [26,70].

No differences were found in the 6-month postoperative visual acuity between phakic patients and pseudophakic patients operated on with DMEK. However, an 11% additional risk of pupillary block was reported in phakic patients operated on with DMEK compared with pseudophakic patients [110].

A recent study has shown that the preoperative lens status did not influence the visual result after DMEK [26]. The preservation of the lens is preferable if it is clear when an endothelial keratoplasty is needed. The rate of re-bubbling does not differ whether the DMEK is performed in combination with cataract surgery and on a pseudophakic or phakic eye [26].

However, the endothelial cell loss is greater in the triple procedure compared with non-combined endothelial keratoplasty at 1 month (35% ± 15 versus 25% ± 16%) and 1 year (41% ± 16% versus 33% ± 13%) [95]. This study did not report any difference in the postoperative visual acuity and the re-bubbling rate between the two types of procedures.

#### 8.1.3. Cataract Surgery After Endothelial Keratoplasty

In a series of 106 eyes operated on with DMEK with a preserved lens, only 5% of eyes needed cataract surgery an average of 9.2 ± 3.7 months after phacoemulsification. No graft detachment was observed, and the visual acuity was at least 6/10 between 6 and 12 months after surgery. The average endothelial cell density decreased from 1535 ± 195 cells/mm^2^ before phacoemulsification to 1158 ± 250 cells/mm^2^ after surgery [111]. However, this study only included five patients, with two patients showing severe glaucoma with tube drainage, which is known to increase endothelial cell loss [112]. This can explain the 25% endothelial loss in this series compared with an expected annual loss of 6% after DMEK [113].

Following cataract surgery in one eye with a history of DSAEK, an average endothelial cell loss of 16 ± 144 cells/mm^2^ was reported at 13 months post-surgery in a series of 60 eyes [109].

### 8.2. Contribution of Imaging Technologies in Combined Surgery Decision

#### 8.2.1. OCT

The central corneal thickness is not the best predictive factor but remains useful in deciding whether to perform combined surgery.

A study has nonetheless evaluated that cataract surgery among patients with Fuchs’ dystrophy and an ECC < 640 µm presented a low risk of corneal decompensation and an improvement in visual acuity [114].

#### 8.2.2. Corneal Densitometry

A study has elaborated a score of progression risk towards endothelial failure after cataract surgery in Fuchs’ dystrophy eyes. The main risk factors were corneal backscattering in the anterior layer between 0 and 2 mm from the corneal apex and an increase in the central corneal thickness [115].

The optimal cutoff points to predict progression to endothelial keratoplasty were >763 scatter units for corneal backscattering in the anterior layer between 0 and 2 mm from the corneal apex with a sensitivity of 89% and >645 µm for pupillary corneal thickness with a sensitivity of 56%.

#### 8.2.3. In Vivo Confocal Microscopy

This device allows us to obtain images of the different layers of the central cornea with a magnification of ×500 and a lateral resolution of 0.6 μm/pixel. The backscattering of the cornea and that of the anterior, mid, and posterior stroma have been calculated semi-automatically with a specific algorithm [116].

A corneal backscattering in the basal layer of the corneal epithelium measured by confocal microscopy, with a threshold of 1894 units, is a predictive factor of the need for an endothelial keratoplasty after cataract surgery in patients with Fuchs’ dystrophy [105].

### 8.3. Refraction and Hyperopic Shift

The advantage of DMEK compared to DSAEK in the triple procedure is that a better refractive precision is obtained as the DMEK graft is finer and, therefore, only modifies the keratometry slightly. The DSAEK in itself could induce a higher hyperopic shift as the graft is thicker at the periphery than in the center. A study from the American Academy of Ophthalmology (AAO) reported a hyperopic shift for DMEK varying from +0.03 to +1.2 diopters with an average of +0.3 diopters [117]. Another study from the AAO on DSAEK reported a hyperopic shift for DSEK varying from +0.7 to +1.5 diopters with an average of +1.1 diopters [118]. The hyperopic shift is, on average, approximately +0.50 to +0.75 diopters for DMEK [109,110], whereas in DSEK, the hyperopic shift varies from +0.88 diopters [119] to +1.46 diopters [120]. Twelve months after surgery, there is no significant difference in the hyperopic shift between DSAEK and UT-DSAEK [47].

Comparing UT-DSAEK and DMEK, a study found no significant differences in the postoperative hyperopic shift at 1 year, respectively, +0.58 ± 1.07 diopters [0.13–1.03] and +0.22 ± 1.19 diopters [−0.23 to 0.68] [52].

The hyperopic shift after a triple procedure occurs mainly in the corneas, which are flatter in the center than in the periphery (posterior oblate cornea). These patients exhibit three times more risks of hyperopic shift after combined DMEK and cataract surgery [121].

The SRKT formula is the one giving the lower refractive error between the desired refraction and the postoperative refraction after DMEK (0.73 ± 0.49 D), and Haigis’ formula is the one giving the higher error (0.90 ± 0.40 D) [122].

Taken together, it is advised to target myopic refraction between −1.0 and −1.5 diopters using the SRKT formula, and when factor Q is positive, to add −0.5 diopters to the power of the selected implant [122].

### 8.4. Cataract Surgery Technique in Fuchs’ Dystrophy

#### 8.4.1. Type of Incision

Scleral incisions do better than clear corneal incisions in terms of endothelial cell loss in the context of cataract surgery in patients with normal endothelial function [123].

A comparative study showed that the average loss of endothelial cells at 6 months and 2 years was significantly higher for a corneal incision of 3.5 mm compared with a scleral incision of 3 mm [124].

#### 8.4.2. Type of Cataract Surgery

A prospective case study did not find any difference in the postoperative CCT or the endothelial cell count between femtosecond laser-assisted cataract surgery and standard phacoemulsification [125].

However, a recent retrospective study comparing the modes of cataract surgery in patients with Fuchs’ dystrophy found a lower endothelial cell loss in the femtosecond laser group (120 ± 435 cells/mm^2^) compared with the phacoemulsification group (346 ± 420 cells/mm^2^) [126]. This can result in a lower risk of endothelial decompensation with the femtosecond laser-assisted cataract surgery technique.

Standard surgery remains recommended for combined endothelial keratoplasty and cataract surgery. Most femtosecond laser devices require a transfer of the patient between the two operating rooms, and its benefit in cataract surgery remains debated in the literature [127].

## 9. Discussion

Today DMEK is the preferred technique in endothelial keratoplasty. DSAEK and, more specifically, UT-DSAEK remain nonetheless a quasi-equivalent alternative. In our review, the main advantage of DMEK and nanofine DSAEK compared to DSAEK standards is a quicker visual recovery. The quality of the final vision was not dependent on the thickness or the regularity of the graft.

Fuchs’ dystrophy is a frequent pathology that increases with age and affects women more particularly. When there is corneal edema, an endothelial graft is recommended in order to prevent the development of intrastromal fibrosis.

The preoperative visual acuity is a strong marker in taking a decision for surgery, a low visual acuity being linked to a lower visual recovery. Furthermore, the corneal OCT is an indispensable tool for the follow-up of patients with Fuchs’ dystrophy by determining the CCT. It also allows the visualization of the thickening of Descemet’s membrane and the subepithelial fibrosis. The Scheimpflug technique allows one to detect the first warning signs of corneal edema.

A combined cataract-endothelial graft surgery can be performed if a cataract is clinically evident. Simple cataract surgery can be questioned in patients with Fuchs’ dystrophy with an ECC < 640 µm, particularly with a scleral incision to perform phacoemulsification.

One can propose the following criteria in order to recommend an endothelial graft in Fuchs’ dystrophy:

Major criteria:

- A preoperative visual acuity below 20/40.

The 20/40 visual acuity threshold is often chosen because it often corresponds to the visual acuity required for obtaining a driver’s license. This criteria may be revised depending on the patient’s needs. Elderly patients who do not need to drive a car may keep an acceptable quality of life with a visual acuity lower than 20/40. Conversely, younger patients with high visual needs may be candidates for DMEK with less impaired vision. The combination of impaired vision and corneal edema is a logical requirement for keratoplasty. Some surgeons may consider proposing a DMEK in eyes with preserved visual acuity. The rationale would be the presence of symptoms such as halos or impaired contrast sensitivity related to corneal guttae. The level of evidence supporting this approach is currently low, and further studies are needed to determine whether the benefit-risk ratio is in favor of DMEK surgery in such cases.

- A clinical corneal edema.

Minor criteria:

- An important visual impact evaluated by V-Fuchs questionnaire;
- A CCT > 625 µm;
- Ratio thickness center–periphery (4 mm with respect to the center) in OCT at 1.03 ± 0.07 (advanced stage) and 0.95 ± 0.07 (moderate stage);
- At least two out of three indicators in OCT analysis with the Scheimpflug’ technique:
  ○ The loss of parallelism or of the regularity of the isopaches;
  ○ The displacement of the finest point;
  ○ Posterior focal depression;
- An increased corneal densitometry, particularly in the anterior layers between 0 and 2 mm of the corneal apex;
- Other criteria (non-determinant):
- Age;
- Gender;
- Genetic background.

Risk scores could be elaborated in the future to estimate the evolution of the pathology and propose endothelial graft surgery to the patient at the most suitable moment to maximize the benefits with respect to the risks. The decision for surgery must remain an individual one for each patient, using the whole range of extra tests available.

## Figures and Tables

**Table 1:** Preoperative visual acuity depending on the different surgical techniques according to various international studies.

Study	Year	Surgical Indication		Surgical Techniques	Number of Eyes	Preoperative Visual Acuity			
		Fuchs’ Dystrophy (%)	Bullous Keratopathy (%)			LogMar	Snellen	Decimal	<20/40 (%)
Afshari et al. [38]	2006	100	0	PK	546	0.50	20/64	0.31	16.9
Price et al. [39]	2006	90	10	DSEK	200	0.69	20/100	0.2	
Bahar et al. [40]	2008	35.4	64.6	PK	48	1.27 ± 7.5 lines	20/372	0.05	
		41.2	58.8	DLEK	68	1.12 ± 7.4 lines	20/263	0.07	
		50	50	DSEK	16	0.52 ± 1.1 lines	20/66	0.27	
		62.2	37.8	DSAEK	45	0.9 ± 5 lines	20/160	0.12	
Price et al. [41]	2009	85	15	DMEK	60	0.39	20/50	0.4	
Terry et al. [42]	2009	100	0	DSAEK	203	0.49	20/62 [20/2000–20/20]	0.32	
Busin et al. [43]	2013	69.6	30.4	UT-DSAEK	250	0.76 ± 4.9 lines	20/115	0.17	6.9
Monnereau et al. [44]	2014	68.2	31.8	DMEK	275				14.5
Rodríguez et al. [45]	2015	89.2	10.8	DMEK	499				38
Wackrer et al. [46]	2016	100	0	DSEK	100	0.45 ± 1.9 lines	20/63	0.35	
Dickman et al. [47]	2016	100	0	DSAEK	32	0.35 ± 2.2 lines [0.27–0.42]	20/44	0.45 [0.37–0.54]	
		100	0	UT-DSAEK	34	0.37 ± 1.8 lines [0.31–0.43]	20/47	0.43 [0.37–0.49]	
Schlögl et al. [48]	2016	91	9	DMEK	97	0.64 ± 4.1 lines	20/87	0.23	19%
Schaub et al. [24]	2017	100	0	DMEK	160	0.4 ± 1.9 lines	20/50	0.39	
Woo et al. [36]	2019	24.4	75.6	PK	405	1.7 ± 5 lines	20/1000	0.02	
		39	61	DSAEK	423	1.2 ± 6 lines	20/317	0.06	
		63.6	36.4	DMEK	121	0.9 ± 6 lines	20/159	0.12	
Brockmann et al. [49]	2019	100	0	DMEK	108	0.57 ± 2.2 lines	20/74	0.27	
Tourabaly et al. [50]	2019	97.3	2.7	DMEK	38	0.48 ± 3.1 lines	20/60	0.33	
		83.3	16.7	Nanothin DSAEK	18	0.85 ± 5.7 lines	20/141	0.14	
		90.3	9.7	UT-DSAEK	52	0.84 ± 3.8 lines	20/138	0.14	
		96	4	Fine DSAEK (100–150 µm)	25	0.97 ± 4.3 lines	20/186	0.11	
		94.1	5.9	DSAEK (>150 µm)	17	0.76 ± 4.4 lines	20/115	0.17	
Birbal et al. [26]	2020	85.3	14.7	DMEK	799	0.46 ± 3.8 lines	20/57	0.35	40.7
Birbal et al. [51]	2020	89.2	10.8	DMEK	451	0.49 ± 3.9 lines	20/62	0.32	40.1
Dunker et al. [52]	2020	100	0	UT-DSAEK	25	0.31 ± 1.3 lines [0.26–0.37]	20/41	0.49	
		100	0	DMEK	29	0.37 ± 1.8 lines [0.30–0.44]	20/47	0.43	

DMEK: Descemet membrane endothelial keratoplasty. DSAEK: Descemet stripping automated endothelial keratoplasty. PK: penetrating keratoplasty. LogMAR: logarithm of the minimum angle of resolution. UT-DSAEK: ultra-thin Descemet stripping automated endothelial keratoplasty.

**Table 2:** Graded classification of Fuchs’ dystrophy with a slit lamp.

Stage of the Disease	Grade	Criteria (Central guttae/Corneal Paracentral)
Not affected	0	Absence of guttae
Intermediary	1	1 to 12 non-merging guttae
	2	More than 12 non-merging guttae
	3	Confluent guttae over 1 to 2 mm
Severe	4	Confluent guttae over 2 to 5 mm
	5	Confluent guttae > 5 mm
	6	Confluent guttae > 5 mm with epithelial edema/visible stroma

**Table 3:** Preoperative and postoperative central corneal thickness and reduction in central corneal thickness in the largest series of studies on DMEK.

Study	Year	Surgical Indications		Surgical Techniques	Number of Eyes	Preoperative CCT (μm)	1-Month Postoperative CCT (μm)	Mean Reduction in CCT (%)
		Fuchs’ Dystrophy (%)	Bullous Keratopathy (%)					
Afshari et al. JAMA Ophtalmology [38]	2006	100	0	PK	259	681 [539–940]	NC	NC
Price et al. Ophtalmology [41]	2009	85	15	DMEK	60	656 [506–1030]	528 [424–678]	19.5
Rodríguez-Calvo-de-Mora et al. Ophtalmology [45]	2015	89.2	10.8	DMEK	499	667 ± 92	525 ± 46	20 ± 11
Wackrer et al. Ophtalmology [46]	2016	100	0	DSEK	100	696 ± 60	656 ± 48	6
Dickman et al. Ophtalmology [47]	2016	100	0	UT-DSAEK	34	643 ± 62 [621–665]	NC	NC
		100	0	DSAEK	32	641 ± 64 [618–664]	NC	NC
Schölg et al. AJO [48]	2016	91	9	DMEK	97	644 ± 67	538 ± 61	18
Schaub et al. AJO [60]	2017	10	0	DMEK	160	596 ± 53 [395–808]	527 ± 56 [430–647]	12.5
Brockmann et al. Current Eye Research [49]	2019	100	0	DMEK	108	660 ± 84	535 ± 82	19
Tourabaly et al. British Journal of Ophtalmology [50]	2019	97.3	2.7	DMEK	38	622 ± 58	529 ± 48	15
		83.3	16.7	Nanofine DSAEK	18	673 ± 62	550 ± 50	18
		90.3	9.7	UT-DSAEK	52	661 ± 77	597 ± 51	10
		96	4	Fine DSAEK (100–150 µm)	25	657 ± 83	622 ± 39	6
		94.1	5.9	DSAEK (>150 µm)	17	715 ± 110	681 ± 42	5
Birbal et al. AJO [26]	2020	85.3	14.7	DMEK	799	687 ± 144	522 ± 54	22
Birbal et al. Cornea [51]	2020	89.2	10.8	DMEK	425	667 ± 192	525 ± 46	20 ± 11

AJO: *American Journal of Ophthalmology*. DMEK: Descemet membrane endothelial keratoplasty. DSAEK: ultra Descemet stripping automated endothelial keratoplasty. JAMA: *Journal of the American Medical Association*. PK: penetrating keratoplasty. CCT: central corneal thickness. UT-DSAEK: ultra-thin Descemet stripping automated endothelial keratoplasty. μm: microns.
